# Digital support principles for sustained mathematics learning in disadvantaged students

**DOI:** 10.1371/journal.pone.0240609

**Published:** 2020-10-23

**Authors:** Frank Reinhold, Sarah Isabelle Hofer, Stefan Hoch, Bernhard Werner, Jürgen Richter-Gebert, Kristina Reiss

**Affiliations:** 1 Heinz Nixdorf-Chair of Mathematics Education, TUM School of Education, Technical University of Munich, Munich, Germany; 2 Institute for Mathematics Education (IMBF), Freiburg University of Education, Freiburg, Germany; 3 Chair of Education and Educational Psychology, Faculty of Psychology and Educational Sciences, University of Munich (LMU), Munich, Germany; 4 Chair for Geometry and Visualization, Department of Mathematics, Technical University of Munich, Munich, Germany; Lingnan University, HONG KONG

## Abstract

This study addresses the pressing issue of how to raise the performance of disadvantaged students in mathematics. We combined established findings on effective instruction with emerging research addressing the specific needs of disadvantaged students. A sample of *N* = 260 disadvantaged 6th-graders received 4 weeks (15 lessons) of fraction instruction either as usual or evidence-based instruction, with and without digital learning support (i.e., interactivity, adaptivity, and immediate explanatory feedback). To examine the sustainability of effects, we assessed students’ fraction knowledge immediately after the 4 weeks and once again after a period of additional 8 weeks. Generalized linear mixed models revealed that students only benefitted from evidence-based instruction if digital support was available in addition. Digital support principles implemented in evidence-based instruction helped disadvantaged students to acquire mathematics knowledge—and to maintain this knowledge.

## Introduction

Mathematics proficiency is a prerequisite not only for learning in diverse subject areas but also for many fast-growing jobs [1, 2]. Given its importance for educational success and career options, the number of students performing on a low level on both international and state tests of mathematics proficiency in countries around the world is alarming [3–5]. About 20% of the 15-year-olds in PISA demonstrate mathematical competence that is below the baseline level considered necessary for full participation in society—and this number is broadly stable from 2006 to 2018 [5]. A considerable number of students does not acquire *sustainable knowledge* (i.e., knowledge that is maintained after instruction) about basic mathematical principles in their mathematics classes [6]. Generating sustainable learning effects that endure over a longer period of time after instruction—hopefully forever—is challenging and, as evidenced in various standardized large-scale assessments, often failing [7, 8].

It is particularly the group of disadvantaged students with limited resources in their surrounding that struggle most to develop sustainable mathematics knowledge [9]. Not only the family’s but also the school’s socioeconomic status considerably influence a student’s accomplishments in mathematics, with the two being highly related [10, 11]. For instance, in the tracked school system of Germany, attendance of the lowest school track *Hauptschule* is highly confounded with below-average socioeconomic status [12, 13]. Not surprisingly, this group of students regularly performs on a lower level on national and international mathematics assessments than students from the highest track, with differences at the end of Grade 6 as high as *d* = 2.68 [14]. Half of all students attending German *Hauptschule* performed below the baseline level in PISA 2012 [15]. These students represent a sample of disadvantaged learners urgently in need of support.

Understanding of fractions and rational numbers is a particularly challenging but at the same time a fundamental part of mathematical competence [16]. This key facet of mathematical literacy is predictive of later achievement in mathematics such as algebra [17] and considered a gatekeeper of higher mathematics [18]. One common source of errors handling fractions is the natural number bias. It describes the tendency to overgeneralize concepts working for natural numbers—e.g., the counting scheme—to rational numbers and fractions [19–21]. To overcome the natural number bias, effective mathematics instruction hence supports learners to restructure existing knowledge to integrate new conceptual knowledge on rational numbers [22, 23]—e.g., that multiplication can yield larger *or* smaller numbers [24].

Over the past few decades, research in mathematics education, educational psychology, and cognitive science has identified principles that have the potential to promote the understanding of fractions. Effective fraction instruction should address the natural number bias [25], stimulate conceptual change [23], and promote the development of rational number concepts (such as representation, density, size, and operation; see [20] for an overview). This can be achieved by creating tasks that induce cognitive conflict and the desire to alter existing concepts of numbers [26], by working with different representations of fractions [22], or by comparing, self-explaining and exploring solution strategies [27]. Instructional principles have repeatedly been found to produce higher learning effects than traditional methods for the average student—yet, disadvantaged students might need extra support in order to gain sustainable learning effects. These instructional principles are effective since they require learners to use existing knowledge to actively process the information provided, thereby constructing new integrated knowledge [28]. Yet, if prior knowledge or the capabilities and motivation to integrate new knowledge into existing knowledge structures are lacking, these cognitively activating instructional methods are unlikely to produce significant knowledge gains [22]. Instruction involving representational gestures, physical movement, and systematic feedback, for instance, has proven effective in sixth graders with mathematics difficulties [29]. Students with non-beneficial cognitive potential—i.e., with low content-specific and strategic prior knowledge, motivation, or less developed reasoning abilities—seem to particularly profit from tablet-based mathematics instruction featuring animations, adaptive tasks, and guidance during practice [22, 30]. Although not restricted to digital environments, new technologies open up a broad range of promising possibilities to help educators implement additional learning support for disadvantaged students (Table 1). These types of learning support could function as catalysts that allow those students to profit from cognitively activating instructional materials and activities by scaffolding the integration of new knowledge (e.g., providing hints, visualizing processes that learners would otherwise have to imagine themselves, ascertaining learning in the zone of proximal development). Support that is tailored to the learners’ preconditions should reduce the complexity of the learning situation just enough to allow active processing of information without overload [31, 32]—which might be of specific importance for low-achieving students [22]. If then new knowledge is integrated into existing knowledge structures (which often also have to be restructured in this process) sustainable accessible knowledge is constructed.

**Table 1:** Principles to support learning in disadvantaged students.

Principle	Examples
**Interactivity** [22, 33, 34]	exploratory tasks involving congruent gestures (e.g., cutting through pizza in terms of swiping over a touchscreen or filling tape diagrams via finger movement to represent a specific part of the whole)
	interactive diagrams (e.g., altered iconic representation of a given fraction to demonstrate what happens, when the enumerator or the denominator is changed, or the fraction is raised)
**Adaptivity** [35–38]	adaptive adjustment of task difficulties (e.g., students have to complete sets of tasks within a certain predefined difficulty level before they are allowed to proceed to the next difficulty level; tasks answered incorrectly have to be repeated until a heuristically determined threshold of correct answers is reached)
	graded assistance during problem-solving (e.g., students can choose to get constructive hints for solving a problem)
**Immediate explanatory feedback** [38–42]	feedback for an incorrect answer is given based on students’ answers (e.g., the correct solution is shown together with the student’s answer)
	feedback for an incorrect answer is given based on an algorithm choosing an appropriate strategy for a given problem (e.g., for comparing 7/6 vs. 8/9, benchmarking to 1 is suggested by the algorithm, while for comparing 4/9 vs. 3/5, benchmarking to 1/2 is suggested)

In this classroom intervention study, we investigated how disadvantaged students can be supported *during* classroom instruction to *maintain* newly-acquired knowledge about fractions *after* classroom instruction. To that end, we combined what we know about effective fraction instruction: we focused on a fraction curriculum based on evidence-based preparation of the learning content (see “Material” section)—proven effective without additional digital learning support in ongoing research in mathematics education [43–45]. Regarding digital support principles, we draw on our recent study [22] that yielded reliable empirical evidence that those principles given in Table 1 implemented as features of educational technology (i.e., interactive and adaptive scaffolds in e-textbooks, see “Material” section) lead to higher-developed fraction concepts in disadvantaged students *immediately after* classroom instruction than learning in paper-based scenarios without those digital support principles—while utilizing those digital support principles showed no significant effect for higher achieving students.

Of specific interest in this study is whether the benefits of those digital support principles *maintain* after formal instruction in school classrooms. To examine this sustainability of effects, we draw on the *identical sample* of disadvantaged students reported in [22]—and assessed students’ knowledge immediately after the intervention (posttest data reported in [22]) and once again after an additional period of eight weeks (i.e., twice the intervention time) without any further instruction on fractions in between (follow-up test data reported solely in this study).

## Materials and methods

The studies involving human participants were reviewed and approved by the Bavarian ministry of education, Germany, reference X.7-BO5106/141/8, and the responsible local education authority “Staatliches Schulamt München für Mittelschulen”, reference SchRIII/Erh106/1—including ethics and data privacy approval. Written informed consent to participate in this study was provided by the participants’ legal guardian/next of kin.

The present classroom intervention study was conducted as a cluster randomized controlled trial with classrooms as clusters on the topic of basic fraction concepts. It followed a pre-post-follow-up constructive research strategy [46], which allowed for isolating effects of digital support principles to support learning in disadvantaged students (Table 1) via graduated treatment in two experimental groups (Scaffolded Curriculum, and Curriculum) and one control group (Traditional): the Scaffolded Curriculum group received the full treatment (i.e., evidence-based fractions curriculum, see “Material” section, *with* digital support principles realized in an e-textbook on iPads), the Curriculum group received a reduced treatment (i.e., evidence-based fractions curriculum *without* digital support principles in a paper-based book), and the Traditional group received instruction on the *same topic* of fractions prepared by their teachers. Given this design, effects between the Scaffolded Curriculum group and the Curriculum group result from digital support principles (Table 1), while effects between the Curriculum group and the Traditional group result from an evidence-based fractions curriculum. The comparison of these two experimental conditions allowed us to examine the hypothesis that disadvantaged students need extra support in addition to evidence-based mathematics instruction to both *acquire* (as already shown in [22]) and to *maintain* (as the main interest of this study) knowledge about fractions.

### Sample

The study included 260 6th-grade students (42% female) from 15 classrooms from the German (Bavaria) public-school track *Hauptschule* (lowest track, below average grades in mathematics and language in grade 4). As described above, this sample is identical to the low-achieving sample reported in [22]. Students from this lowest school track on average reach only moderate to low mathematics achievement in standardized tests across different grade levels [14, 15] and come from families with, on the average, lower socioeconomic status than other students [12, 13, 47]—underpinning our operationalization of disadvantaged students that need specific assistance when to develop and maintain mathematical knowledge.

It is noteworthy that fractions are formally introduced in grade six in the mandatory German (Bavarian) curriculum, so that no formal knowledge about fractions acquired in school has to be expected by 6th-graders in this study *before* the intervention.

### Material

We created an evidence-based fractions curriculum to teach basic fraction concepts—i.e., *i*. the part-whole concept with one and many wholes using iconic, continuous, discrete as well as symbolic representations [43]; *ii*. fraction magnitude [21]; *iii*. expanding and simplifying fractions, i.e., divisions becoming more refined or coarser [44, 48]; *iv*. fractions on the number line [49]; *v*. fractions representing more than one whole and mixed numbers [45]; and *vi*. the comparison of the size of two fractions based on different strategies [21]. We compared our curriculum to the mandatory curriculum for grade six in Bavaria, Germany, making sure that students within all three groups were exposed to the same content. In general, our evidence-based curriculum differs from traditional approaches by focusing on conceptual rather than procedural knowledge of fractions. Here—in line with a commonly agreed-upon distinction between these types of knowledge [43, 45, 50–52]—we see *procedural knowledge of fractions* necessary to operate with fractions in symbolical representation with no required transition between representations, and *conceptual knowledge of fractions* necessary to operate with iconic representations of fractions or to switch between non-symbolic and symbolic representations [22]. Thus, our curriculum allows for transitions between a variety of non-symbolic and symbolic representations of fractions, provides intuitive pathways to the core fraction concepts given above, and makes sure students explore non-symbolic fractions before more formal representations (for a more detailed analysis of the content with regards to evidence-based mathematics instruction, also refer to [22]).

**Fig 1:**
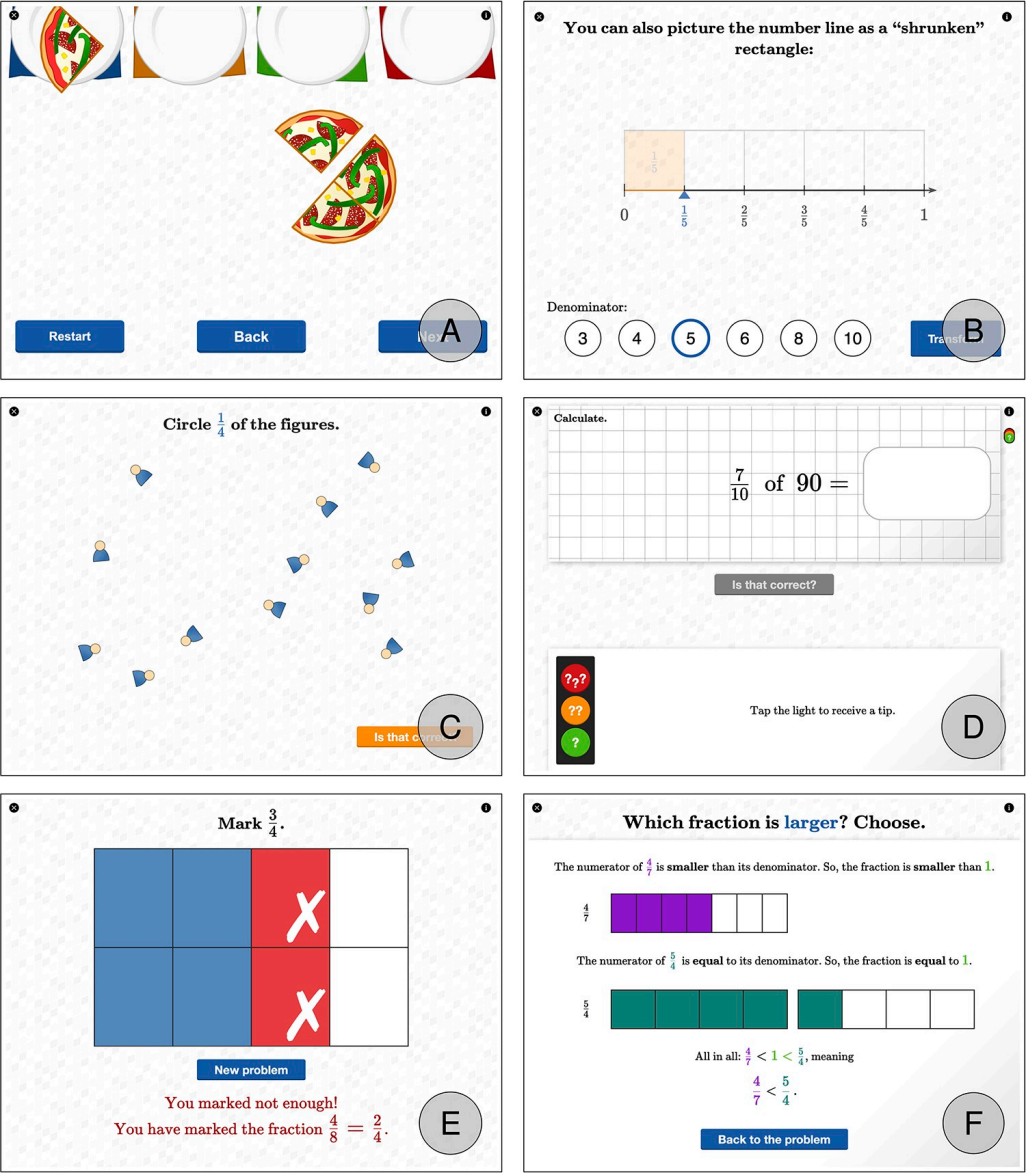
Technological implementation of digital support principles within the e-textbook. (A) Exploratory task involving congruent gestures, i.e., cutting through pizza in terms of swiping over a touchscreen. (B) Interactive diagram showing the conceptual idea of the number line as a shrunken tape diagram—here displayed using overlay technique. (C) Task with adaptive adjustment of task difficulties that represent difficulty generating factors, i.e., number of items equals denominator in level 1, number of items is twice or thrice the denominator in level 2. (D) Task offering graded assistance during problem-solving which students can access by tapping on the traffic lights. (E) Feedback for an incorrect answer based on students’ wrong answers. (F) Feedback for an incorrect answer based on an algorithm choosing an appropriate strategy for the comparison of two fractions.

The Scaffolded Curriculum group worked with an e-textbook on iPads designed within the iBooks Author framework and CindyJS as the programming environment for the interactive content [53]. This e-textbook transported our elaborated fractions curriculum utilizing the principles to support learning in disadvantaged students (Table 1) in 90 interactive exercises, i.e., widgets (for a more detailed presentation of the technological implementation, also refer to [22, 53]). A detailed overview of the technological implementation of the digital support principles described in Table 1 within the e-textbook is depicted in Fig 1. The interactive content is embedded in the e-textbook as widgets, allowing for a rather typical textbook structure, i.e., page numbers, text passages, numbered tasks, headlines, etc.

A paper-based copy of the e-textbook was used in the Curriculum group. The paper-based version differed from the e-textbook only in terms of the absence of digital learning support. Both the e-textbook [54] and the paper-based version [55] are available online under a CC-BY 4.0 license.

### Instruments

In order to control for effects of prior knowledge of fractions, a pretest was conducted before the intervention (10 items; internal consistency of McDonald’s Omega ω = 0.82; full independent double coding with an inter-rater reliability of 0.85 ≤ κ ≤ 0.99). The original German version of the test instrument is available online [56], and an English translation of the instrument is given in the S1 File. As the topic of fractions is introduced only in grade six in the legal curriculum in Germany (Bavaria), we decided not to use the posttest instrument to assess students’ prior knowledge of fractions (as one must expect a floor effect before the intervention), but to design an instrument assessing students’ knowledge on every-day fractions (e.g., 1/2 or 3/4)—which was used as a covariate in the analysis of achievement after the intervention.

To measure basic fraction knowledge, a second test instrument was developed (16 dichotomous items; internal consistency of McDonald’s Omega ω_Post_ = 0.68 in the posttest, and ω_FollowUp_ = 0.63 in the follow-up test; full independent double coding with an inter-rater reliability of 0.93 ≤ κ ≤ 0.99). Here, items focus on the six fraction topics given above. The developed instrument was used both for the posttest and for the follow-up test. All originally German items from this instrument can be found online [56], and an English translation of the instrument is given in the S2 File.

### Procedure

We conducted a pilot study to ensure the quality of the instruments (pretest *n* = 142, posttest *n* = 247) and the e-textbook (*n* = 32) one year before the main study.

The present study was conducted at the beginning of the school year 2017/2018. After review and positive evaluation by the Bavarian ministry of education and the responsible local education authority (including ethics and data privacy approval), a total number of 44 schools were asked to take part in the study on a voluntary basis and 25% of the schools answered this invitation, including eight positive and three negative responses. In voluntary agreement with the schools’ principals and the classroom teachers, students and their parents were informed about the aims and goals of the study. Taking part in the written tests was voluntary for all students, and with informed consent of them and their parents. Only after teachers agreed in taking part in the study, whole classes were randomly assigned to one of the three groups.

The intervention took place within the first 15 mathematics lessons of the school year, covering fractions only. The Curriculum group (*n* = 71) received evidence-based fraction instruction structured by the paper-based book. It focused on transitions between non-symbolic and symbolic representations of fractions, provided intuitive pathways to rational number concepts, and required learners to compare, self-explain and explore [22]. The Scaffolded Curriculum group (*n* = 107) learned with the same evidence-based fraction instruction, however, implemented within an e-textbook on iPads [22, 53]. The e-textbook allowed for digital learning support including hands-on activities, tutoring with adaptive task difficulty, and individual feedback (Table 1). The Traditional group (*n* = 82) attended business-as-usual mathematics classes and teachers were free to choose their method of introducing fractions. In all three groups, the same fraction topics had to be taught during the 15-lesson study period.

**Fig 2:**
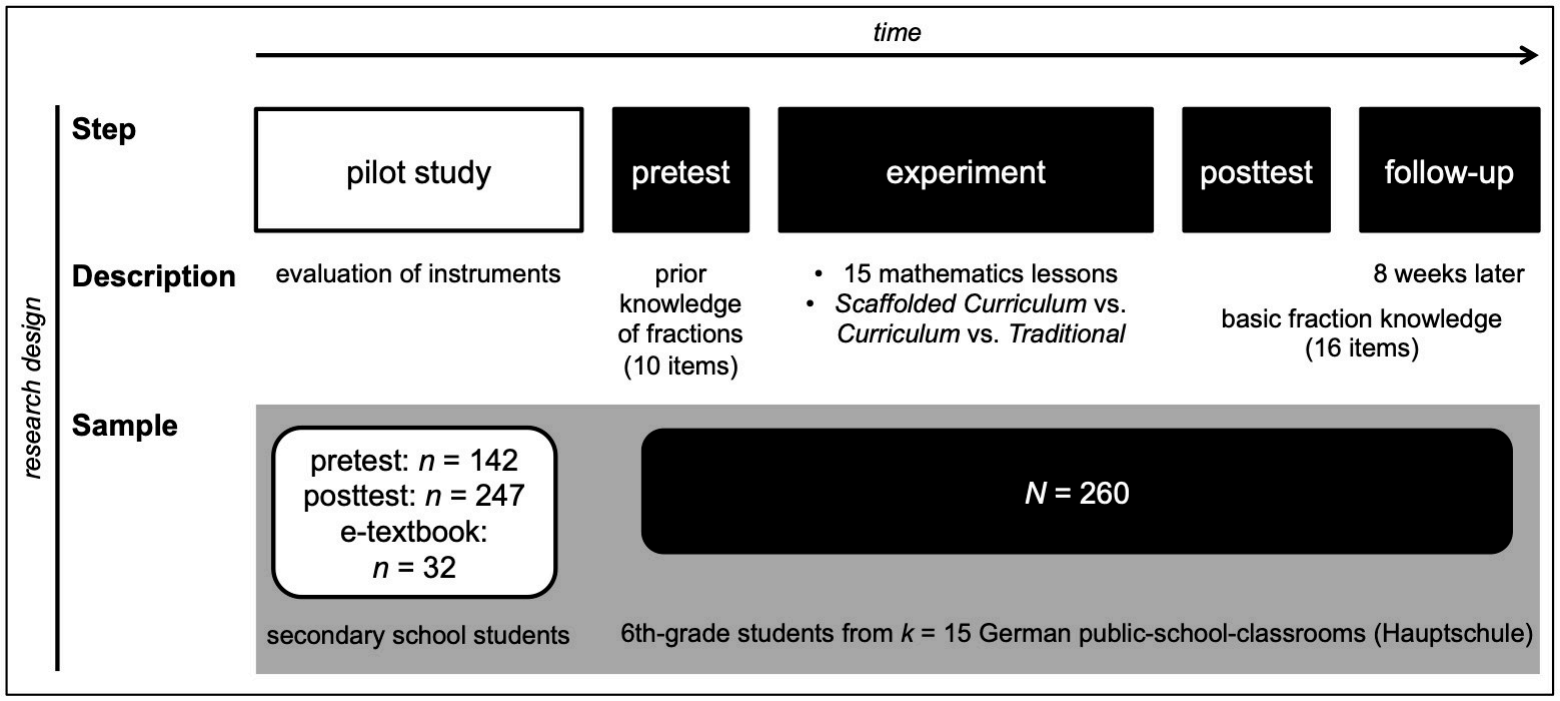
Overview of the study design.

In order to control for effects of prior fraction knowledge, the pretest (15 minutes) was conducted before the first lesson. We assessed fraction knowledge in the posttest immediately after 15 lessons, and eight weeks after the posttest (56 ± 14 days) in the follow-up test (20 minutes). The complete research design is summarized in Fig 2.

### Implementation check

The 15 participating teachers (Scaffolded Curriculum, Curriculum, and Traditional) took part in the same 90-minute teacher training before the intervention. Each teacher received an 18-page booklet with information on the research questions, the educational objectives, and the content to be taught during the 15 lessons.

We conducted structured and formal interviews with all 15 teachers after the intervention to ensure that they covered all topics, did not differ in total instruction time, and used the material provided for the Scaffolded Curriculum group and the Curriculum group as intended. Relying on the interviews, we considered the implementation adequate if and only if …

- Traditional group: … the content listed above was completely addressed during the 15 lessons.
- Curriculum group: … the content listed above was completely addressed during the 15 lessons; our exploratory introductions were used to start with new topics; all 90 tasks (i.e., the printed version of the widgets in the e-textbook) were worked on at least once.
- Scaffolded Curriculum group: … the content listed above was completely addressed during the 15 lessons; our exploratory introductions were used to start with new topics while using the e-textbooks interactive content; all 90 widgets were worked on at least once with recourse to the e-textbooks scaffolds.

The teachers’ statements indicated that all study conditions were implemented as intended. In addition, informal interviews with all 15 teachers were conducted at the follow-up test to ensure that the teachers did not teach the content of the intervention between posttest and follow-up test—which was not the case.

### Data and statistical analysis

A generalized linear mixed model (GLMM) was used to estimate differences between the three intervention groups in answering the test items. For the aim of the current study, they have several advantages over other statistical approaches, i.e., handling of unbalanced designs, as well as nested data structures, and dichotomous data [57–59]. We conducted the analysis in *R* (R version 3.6.2).

The Null model contains only a fixed effect for Time (0 = posttest, 1 = follow-up test), estimating an overall effect of the two different time points on students’ ability to answer items regarding fraction concepts. In the Full model, fixed effects for specific control variables—i.e., Prior Knowledge (pretest result, standardized at the sample), and Gender (-0.5 = female; 0.5 = male)—are added. To estimate effects of the intervention, a fixed effect for Intervention Group (baseline: Scaffolded Curriculum) is added, predicting differences in the posttest. Differences between the three intervention groups in the follow-up are represented in the Time × Intervention Group interaction effect. To control for the variance in the time between posttest and follow-up in the 15 different classrooms, a Time × Delay (number of days between posttest and follow-up, standardized at the sample) interaction effect is added. As the effect of Delay is not meaningful for estimating posttest results, the main effect of Delay is omitted in the Full model. Both the Null model and the Full model allow for a Student, a Classroom, and an Item random intercept—to account for the nested data structure (students clustered in classrooms) as well as difference in item difficulty and student ability. Data used for the analysis is given in the S3 File. We used the *lme4* package (R package version 1.1–21) and the *lmerTest* package (R package version 3.1–1) for the analysis. Post-hoc contrasts between the groups were estimated using the *emmeans* package (R package version 1.4.5).

Estimates are given as Odds Ratios, reflecting unique changes in the estimated probability to answer an average item in the test correct, when a categorial predictor changes from the baseline to another level, or when a metric predictor is 1 SD above the sample mean. Odds ratios and their confidence intervals were calculated using the *sjPlot* package (R package version 2.8.3).

As students were nested in classrooms and classrooms were randomly assigned to one of the three intervention groups, the Proportion Change in Variance [PCV, see 59, 60] in the Classroom random intercept from the Null model to the Full model is of specific interest for this study. It estimates the change in random (i.e., unexplained) variance between the classrooms, when study-related predictors are added to the model.

For less than 4% of the sample (*n* = 2 students from the Scaffolded Curriculum group and *n* = 7 students from the Curriculum group) no pretest data was available. These missing values for prior knowledge of fractions were estimated using multivariate imputation by chained equations—imputing a random sample from the observed values utilizing the *mice* package (R package version 3.8.0). Furthermore, power of the relevant effects were estimated via Monte Carlo simulation utilizing the *simr* package (R package version 1.0.5).

## Results

The groups showed no significant differences in terms of prior knowledge, *F*(2, 256) = 1.591, *p* = 0.206 (Table 2). Furthermore, girls and boys did not differ significantly in term of prior knowledge before the intervention, *F*(1, 256) = 1.828, *p* = 0.178 (Table 2). To account for individual differences, both prior knowledge and gender are considered as control variables in the main analysis.

**Table 2:** Pretest outcomes for the total sample, female and male students, as well as students from the three groups.

**Total sample**	**N**	**M**	**SD**
	260	2.28	2.02
**Gender**	**N**	**M**	**SD**
female	110	2.21	2.04
male	150	2.33	2.01
**Intervention groups**	**N**	**M**	**SD**
Scaffolded Curriculum	107	2.55	2.18
Curriculum	71	2.17	1.96
Traditional	82	2.02	1.81

*Note*. N = Sample size, M = Mean, SD = Standard deviation.

There was a significant effect of prior knowledge on posttest fraction knowledge (Table 3). Students yielding pretest scores 1 SD above the sample mean were 23% more likely to answer an average item in the posttest correctly. Prior fraction knowledge hence helped to acquire new knowledge. Yet, students’ gender did not affect the posttest outcome significantly (Table 3).

**Table 3:** Estimates of the generalized linear mixed models used to predict the likelihood for a correct answer in the posttest and the follow-up.

	Null Model			Full Model		
Fixed effects	OR	CI	p	OR	CI	p
*Control Variables*						
Prior Knowledge				1.23	[1.13, 1.35]	< 0.001
Gender				1.00	[0.84, 1.19]	0.990
**Intervention groups [baseline: Scaffolded Curriculum]**						
Scaffolded Curriculum → Curriculum				0.66	[0.47, 0.91]	0.012
Scaffolded Curriculum → Traditional				0.50	[0.37, 0.69]	< 0.001
**Longitudinal effect [Posttest to Follow-Up]**						
Time	0.98	[0.88, 1.09]	0.721	0.98	[0.83, 1.16]	0.818
Time × Curriculum				1.03	[0.79, 1.35]	0.839
Time × Traditional				0.97	[0.74, 1.26]	0.800
Time × Delay				0.94	[0.83, 1.05]	0.274
**Random effects**	**Var**			**Var**	**PCV**	
Student (*N* = 260)	0.32			0.28	11.81%	
Classroom (*N* = 15)	0.16			0.03	81.41%	
Item (*N* = 16)	1.49			1.49	0.57%	
**Model characteristics**						
Observations	7888			7888		
AIC	8441			8421		

*Note*. OR = Odds ratio, CI = 95% Confidence interval, Var = Variance, PCV = Proportion change in variance in the corresponding random intercept from Null model to Full model [59, 60].

**Fig 3:**
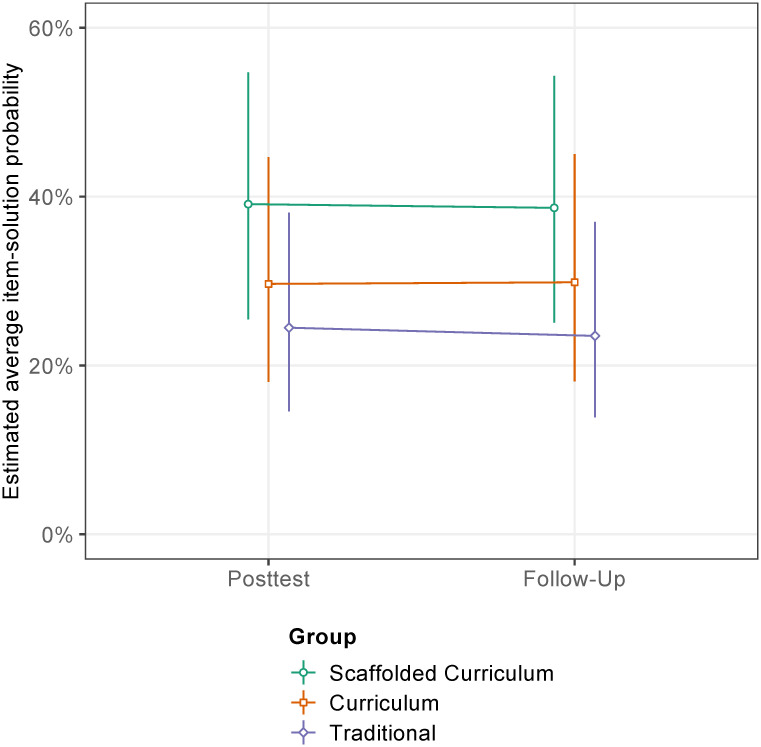
Development of students’ fraction knowledge. Development is shown from the posttest (immediately after the four weeks of instruction during the intervention, i.e., 15 lessons) to the follow-up test (additional eight weeks after the posttest) as estimated average item-solution probabilities, predicted by the generalized linear mixed model. Error bars represent 95% confidence intervals.

We expected digital learning support to be beneficial for disadvantaged students—in addition to evidence-based fraction instruction. In line with this hypothesis, students from the Scaffolded Curriculum group yielded the highest outcomes, both in the posttest and the follow-up (Fig 3, Table 3). Regarding estimated marginal means as a measure for posttest achievement, students from the Scaffolded Curriculum group had a 39.1% probability (95%CI [25.5, 54.7]) to solve an item in the posttest, students from the Curriculum group had a probability of 29.7% (95%CI [18.0, 44.7]), and students from the Traditional group had a probability of 24.5% (95%CI [14.6, 38.1]). Odds ratios represent the change in the likelihood for answering an item correct when comparing students from different experimental conditions and thus are considered estimates of the effects in the present study. Considering all fixed and random effects allowed for in the Full GLMM, Students from the Scaffolded Curriculum group were on average 52% more likely to give a correct answer to an item in the posttest than students from the Curriculum group, *z* = 2.518, *p* = 0.032, and on average 98% more likely than students from the Traditional group, *z* = 4.298, *p* < 0.001. Although students from the Curriculum group had on average a 30% higher estimated probability to correctly answer an item in the posttest than students from the Traditional group, there was no significant difference in posttest outcomes between the Curriculum group and the Traditional group, *z* = -1.492, *p* = 0.295. Thus, digital learning support was decisive for disadvantaged students to benefit from evidence-based fraction instruction. The significant difference between students in the Scaffolded Curriculum group and the Traditional group yielded a statistical power of 99.5%, 95%CI [98.84, 99.84]. The significant difference between students in the Scaffolded Curriculum group and the Curriculum group yielded a statistical power of 76.8%, 95%CI [74.06, 79.38].

Most important for the present study, in none of the three groups, students’ outcomes did not differ significantly between the posttest and the follow-up test in any of the three groups (Scaffolded Curriculum group, *z* = 0.222, *p* = 0.825; Curriculum group, *z* = 0.501, *p* = 0.616; Traditional group, *z* = -0.084, *p* = 0.933). Consequently, the advantage of digitally supported evidence-based fraction instruction was maintained over time without further fraction instruction. Evidence-based fraction instruction *without digital support* was not sufficient to help disadvantaged students to learn more than from traditional instruction.

The *Proportion Change in Variance* [PCV, see 59, 60] in the classroom random intercept can be considered a valid estimate of intervention effects in cluster randomized controlled trials. More than 81% of the variance between classrooms regarded as random in the Null model could be explained in the Full model by including the three experimental groups that evidently caused a large part of the variance between classrooms. This is a promising finding, demonstrating that thoroughly designed instruction can make a big difference across various classrooms differing in classroom composition or teacher characteristics.

## Discussion

### Digital support principles for sustained mathematics learning

Our results yield unique ecologically-valid empirical evidence that digital learning support—implemented in evidence-based fraction instruction—helps disadvantaged students to acquire—and most important to maintain—fraction knowledge in real mathematics classrooms. With regards to the constructive research design, the results suggest that the positive outcome of the Scaffolded Curriculum group—both in the posttest and in the follow-up test—should be ascribed to the implementation of digital support principles, and not the evidence-based fractions curriculum, in the present sample.

Yet, the finding that in all three groups students maintained their respective knowledge level from the posttest to the follow-up test, but differed in the absolute knowledge level gained during the intervention, suggests a more fine-grained analysis of the exact content successfully learned in each condition. Indeed, the low achievement level in the Traditional group suggests that these students only learned most basic content during classroom practice—yet they could maintain this knowledge after eight weeks without additional instruction. When interpreting this result, it should be kept in mind that students in the Scaffolded Curriculum group did also keep their knowledge level without further instruction, yet on a substantially higher level than students from the Control group—which is arguably a more difficult task, underpinning the positive effect of digital support principles for disadvantaged students.

While students with more favorable prerequisites also profit from evidence-based instruction [22], additional support seems to be unnecessary for this group of learners. In turn, instruction based on (traditional, non-digital) principles proven effective for the average student may not be enough for students with unfavorable preconditions for learning. In order to benefit from evidence-based instructional methods, these students require extra assistance. Digital support principles such as interactivity, adaptivity, and immediate explanatory feedback (Table 1) may hence be purposefully deployed in e-learning environments that target disadvantaged or low-achieving students. The list of general support principles, which are not necessarily digital, can be extended to also include motivational support in the form of establishing attainment value or promoting productive attribution [61], and applied to diverse subject areas beyond fractions.

While there is broad empirical evidence that all of our suggested digital support principles mentioned in Table 1 support learning in rather short experimental conditions, there is still a lack in profound research that yields ecologically valid evidence that they also work as expected within real classroom situations [62, 63]. Our study contributes to that research desideratum by providing results from a four-week in-school intervention in a cluster-randomized control trial with classrooms as clusters. Acknowledging the fact that the sample should, therefore, not be considered completely random, we draw on a statistical method that allows for consideration of the present clustered sample structure in the estimation of the standard errors—i.e., GLMM.

### Limitations and future directions

Our operationalization of disadvantaged students as students from the lowest school track of the German public-school system could be considered a potential limitation of this study. However, attendance of this track is not only confounded with lower performance on standardized assessments of mathematics competence [14, 15], but also with lower socio-economic status [12, 13]. Replications of our study using a different operationalization of disadvantaged students could substantiate our findings.

It might also be questioned whether a time period of eight weeks between posttest and follow-up test are enough to assess the sustainability of learning effects. Additional follow-up tests after several months could corroborate our findings. Yet, there is long-established evidence that only eight weeks after instruction about two thirds of successfully learnt information is forgotten [64] and up to 60% of learnt information lasts not longer than one hour [65]. If instruction leads to learning that endures eight weeks without repetition, this can be considered an indication of sustainable effects. In addition, it should be emphasized that the time between posttest and follow-up test in this study is twice the total instruction time during the intervention. Considering the specific sample reported on and the fact that no fractions content knowledge was taught in either of the experimental conditions between posttest and follow-up test, we consider the results in line with the hypothesis of sustainable effects on mathematics learning.

The finding that the students described in [22] and in this study still show large differences in posttest achievement although they received the same intervention suggests that even more basic digital support principles and tools might be needed to aid low-achieving and disadvantaged students in learning mathematics. Such tools could be implemented and tested to compensate for the diverse difficulties students face in learning situations. For instance, automatic word recognition or auto completion algorithms could reduce the cognitive load during reading and writing experienced by students with low proficiency in the instructional language and, thus, free resources for learning. Other examples for digital support that might aid these students may act on the level of motivational and emotional orientations, stimulating students’ self-efficacy while learning demanding tasks such as fractions.

Due to specific circumstances of the present study, students did only have access to the iPads—and therefore to the e-textbook providing digital support for the learning of fraction concepts—in school and during their regular mathematics lessons. It is of specific interest, whether providing access to these support principles for learning outside of school may have additional positive effects. Within these rather informal learning scenarios, students face different boundary conditions when engaging in learning tasks (i.e., learning from home during the global COVID-19 pandemic), so that different effects than in regular school classrooms may be expected—which could be focused on in subsequent studies.

## Supporting information

S1 File English version of the pretest instrument. Please also refer to [56] for the original German version of the instrument, used in this study.
(PDF)

S2 File English version of the posttest and the follow-up test instrument. Please also refer to [56] for the original German version of the instrument, used in this study. Tasks correspond to the itemID in the data (S3 File) in the following way: Task 1a = A20a, Task 1b = A20b, Task 1c = A20c, Task 1d = A20d, Task 1e = A20e, Task 2 = A03, Task 3a = A14a, Task 3b = A14b, Task 4a = A16a, Task 4b = A16b, Task 5a = A15a, Task 5b = A15b, Task 5c = A15c, Task 5d = A15d, Task 6a = A07a, Task 6b = A07b, Task 6c = A07c, Task 7 = A05.
(PDF)

S3 File Data file containing the data relevant for this analysis. studID (anonymized student id, numbers from 401 to 660); claID (anonymized classroom id, numbers from 1 to 15); grp (factor representing group, i.e., Scaffolded Curriculum, Curriculum, Traditional); sexC (student’s gender, (-0.5 = female; 0.5 = male); delayZ (standardized delay between posttest and follow-up, original *M* = 56 days, *SD* = 14); PreZ (standardized pretest outcome, original *M* = 2.28, *SD* = 2.02); itemID (item id, identifying items in the posttest and the follow-up); Time (0 = posttest, 1 = follow-up); Value (0 = wrong answer, 1 = correct answer).
(CSV)
