# Supplementary material for: Digital support principles for sustained mathematics learning in disadvantaged students
Source: PLoS One. 2020 Oct 23;15(10):e0240609. doi: 10.1371/journal.pone.0240609 (PMC7584209; doi:10.1371/journal.pone.0240609)
Supplement: S1 File — Please also refer to [56] for the original German version of the instrument, used in this study. (PDF) [file pone.0240609.s001.pdf]

Task 1. Encircle *three quarters* of the pizzas..

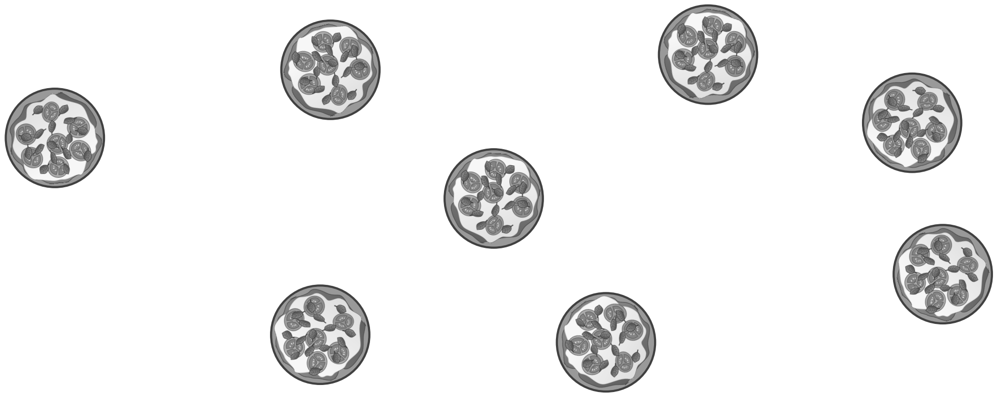

Task 2. Determine what fraction of the beam is colored in gray.

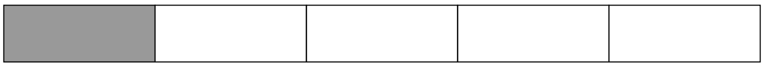

Task 3. A fraction is marked on the number line. Determine it.

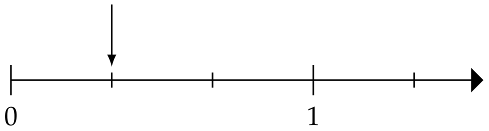

Fraction:

Task 4. Color *two thirds* of the circle in gray.

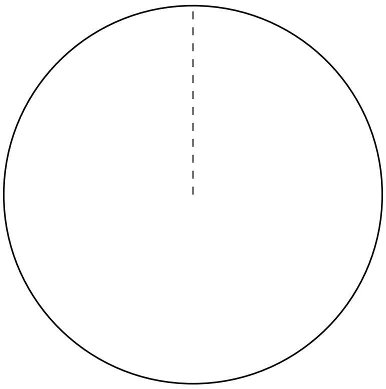

Task 5. Determine how many apples are *two thirds* of 36 apples.

Task 6. Determine what fracion of the circle is colored in gray.

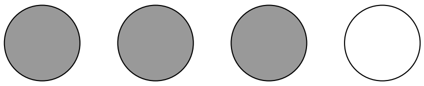

Task 7. Color in *two fifths* of the circle.

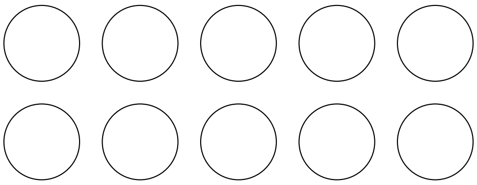

Task 8. Color in *one third* of the beam.

Task 9. Anja bought apples today. The picture shows *three quarters* of her purchase.

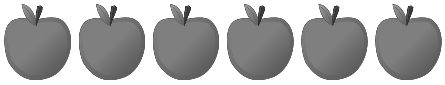

Determine how many apples Anja bought in total.

Task 10. Tick the box with the larger number.

a)	☐ $\frac{2}{5}$ *(2 fifths)*	☐ $\frac{4}{5}$ *(4 fifths)*
b)	☐ $\frac{1}{3}$ *(1 third)*	☐ $\frac{1}{4}$ *(1 quarter)*
c)	☐ $\frac{2}{3}$ *(2 thirds)*	☐ $\frac{2}{5}$ *(2 fifths)*
d)	☐ $\frac{4}{5}$ *(4 fifths)*	☐ $\frac{3}{2}$ *(3 halves)*
