# Supplementary material for: Digital support principles for sustained mathematics learning in disadvantaged students
Source: PLoS One. 2020 Oct 23;15(10):e0240609. doi: 10.1371/journal.pone.0240609 (PMC7584209; doi:10.1371/journal.pone.0240609)

Task 1. Compare the fractions. Fill the box with the correct symbol: „>“, „<“ or „=“

Space for additional calculations:

a) $\frac{3}{7}$ ☐ $\frac{5}{7}$

b) $\frac{4}{5}$ ☐ $\frac{4}{7}$

c) $\frac{3}{9}$ ☐ $\frac{5}{15}$

d) $\frac{20}{21}$ ☐ $\frac{9}{10}$

e) $\frac{15}{13}$ ☐ $\frac{16}{17}$

Task 2. What fraction of the circle is colored in gray? Tick the box.

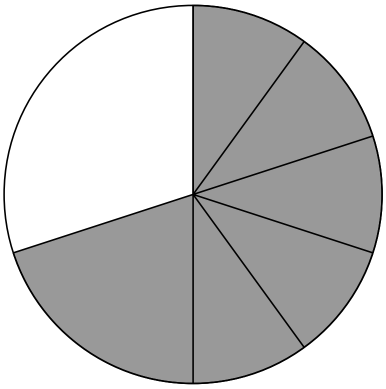

- ☐ $\frac{6}{8}$
- ☐ $\frac{7}{10}$
- ☐ $\frac{7}{12}$
- ☐ $\frac{6}{7}$
- ☐ $\frac{6}{10}$

Task 3. Put the two fractions $\frac{5}{6}$ and $\frac{7}{3}$ on the correct position on the number line.

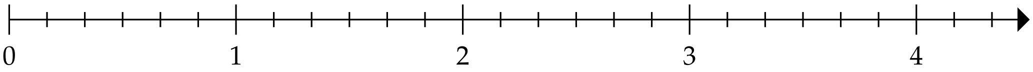

Task 4. Calculate:

a) $\frac{3}{5}$ of $45 =$

b) $\frac{4}{7}$ of $42 =$

Task 5. Is more or less than $\frac{4}{9}$ of the area in the pictures colored in gray? Tick the correct answer.

a) 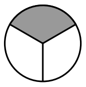 ☐ more than $\frac{4}{9}$ ☐ less than $\frac{4}{9}$

b) 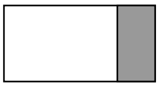 ☐ more than $\frac{4}{9}$ ☐ less than $\frac{4}{9}$

c) 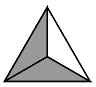 ☐ more than $\frac{4}{9}$ ☐ less than $\frac{4}{9}$

d) 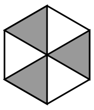 ☐ more than $\frac{4}{9}$ ☐ less than $\frac{4}{9}$

Task 6. Write the missing numbers into the boxes.

a) $\dfrac{3}{\square} = \dfrac{12}{28}$

b) $\dfrac{6}{8} = \dfrac{\square}{20}$

c) $4 = \dfrac{\square}{3}$

Task 7. The depicted fraction should be simplified by 2. Check the correct picture.

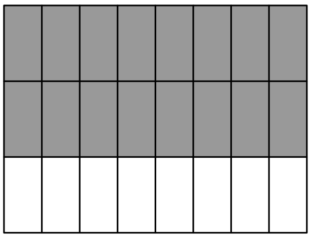

☐ 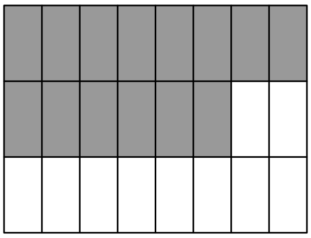

☐ 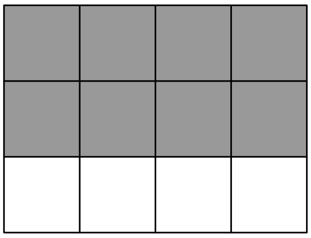

☐ 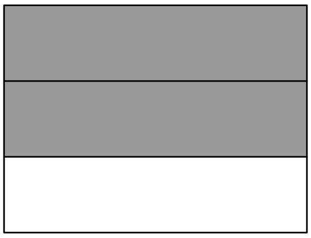

☐ 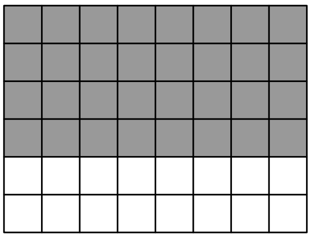

☐ 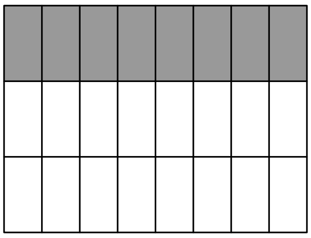

Supplement: S2 File — Please also refer to [56] for the original German version of the instrument, used in this study. Tasks correspond to the itemID in the data (S3 File) in the following way: Task 1a = A20a, Task 1b = A20b, Task 1c = A20c, Task 1d = A20d, Task 1e = A20e, Task 2 = A03, Task 3a = A14a, Task 3b = A14b, Task 4a = A16a, Task 4b = A16b, Task 5a = A15a, Task 5b = A15b, Task 5c = A15c, Task 5d = A15d, Task 6a = A07a, Task 6b = A07b, Task 6c = A07c, Task 7 = A05. (PDF) [file pone.0240609.s002.pdf]
